# Posttraumatic stress disorder among adolescents in Brazil: a cross-sectional study

**DOI:** 10.1186/s12888-021-03062-z

**Published:** 2021-02-05

**Authors:** Joviana Quintes Avanci, Fernanda Serpeloni, Thiago Pires de Oliveira, Simone Gonçalves de Assis

**Affiliations:** 1 Department of Studies on Violence and Health Jorge Careli, National School of Public Health, Oswaldo Cruz Foundation, Avenida Brasil 4036, 700 Manguinhos, Rio de Janeiro, 21040-361 Brazil; 2 International Business Machines Corporation, Rio de Janeiro, Brazil; 3 Neurology Post-Graduate Program, Federal University of the State of Rio de Janeiro, Mariz e Barros 775, Rio de Janeiro, 20270-901 Brazil

**Keywords:** Posttraumatic stress disorder, Violence, Traumatic events, Adolescent, High-school students

## Abstract

**Background:**

The frequency of trauma and different types of violence exposure in urban areas and their effects on the mental health of adolescents in developing countries are poorly investigated. Most information about traumatized young people comes from war scenarios or disasters. This study aimed to determine the prevalence of PTSD in trauma-exposed students in a low-resource city of the state of Rio de Janeiro, Brazil. The effects of sociodemographic and individual and family factors in the development of PTSD were also investigated.

**Methods:**

Through multi-stage cluster sampling, 862 adolescents (*M*age = 15 years old, 65% female) from public and private schools in the city of São Gonçalo were selected for the study. Self-rating structured questionnaires were applied to assess sociodemographic profile, exposure to physical and psychological violence (family, school, community), sexual abuse, social support, social functional impairment, resilience, and posttraumatic stress disorder. The data were grouped in blocks regarding sociodemographic, individual, family, and community variables. For statistical analysis, chi-square, Fisher’s exact test, and logistic regression were performed.

**Results:**

The PTSD prevalence was 7.8% among adolescents. Boys were exposed to significantly higher number of events of community violence, while girls to family violence. The adjusted odds ratio (OR) for PTSD were statistically significant for age (OR, 1.45, [95% CI, 1.043–2.007]), social functional impairment (OR, 4.82, [95% CI, 1.77–13.10]), severe maternal physical violence (OR, 2.79, [95% CI, 0.79–9.93]), psychological violence by significant people (OR, 3.96, [95% CI, 1.89–8.31]) and a high number of episodes of community violence (OR, 3.52, [95% CI, 1.47–8.40).

**Conclusions:**

There was a high prevalence of PTSD within this population associated with exposure to violence. Not only physical, but also psychological violence contributed to PTSD. The results also raise awareness to the differences in life trajectories between boys and girls regarding violence. These differences need to be better understood in order to enable the development of effective preventative interventions. Treating and preventing mental health disorders presents a challenge for countries, especially those with a lower degree of social and economic development and high community violence.

**Supplementary Information:**

The online version contains supplementary material available at 10.1186/s12888-021-03062-z.

## Background

Posttraumatic stress disorder (PTSD) is one of the most common disorders associated with exposure to violence. However, there is limited information about the prevalence of childhood adversities and PTSD among adolescents in low to middle-income countries [1]. Furthermore, little is known about the extent to which individual, family, and community factors relate to PTSD in young people living in regions with high violence levels. Although teenagers exposed to family violence are more likely to experience community violence, these forms of adversity are usually examined separately [2]. Sexual abuse and exploitation, psychological and physical violence and neglect have been registered as the main reasons for intervention from Brazilian social welfare centers for children [3]. In order to address these issues we aim at investigating the prevalence of PTSD in adolescents living in a low-resource urban area and assess the impact of different forms of violence and trauma on PTSD.

Worldwide, the largest proportion of youth live in low and middle-income countries (LMICs) [3]. The World Health Organization estimates that mental health problems affect 10–20% of children worldwide, compared to 12 to 25% of Brazilian children and adolescents [3, 4]. In the Brazilian context, PTSD has become an important public health problem. The prevalence of PTSD in adolescents has come mainly from studies in developed countries. For instance, in the US, a six-month PTSD prevalence of 3.7% for boys and 6.3% for girls among 12–17-year-olds was found [5]. In tornado-affected communities, it was found that 6.7% of the adolescents met diagnostic criteria for PTSD [6]. Studies investigating traumatic stress in low to middle-income countries have focused mainly on disasters and war zones [7, 8]. A small number of PTSD studies in young people have been performed in LMICs, within the social vulnerabilities context with heterogeneous types of traumatic events (i.e. hunger, domestic and community violence) occuring in a cumulative and simultaneous way [3].

In Latin America and the Caribbean, the homicide rate among adolescents is five times higher than the world average. In Brazil, in 2016, homicides were responsible for 49% of deaths among young people aged 15 to 19, representing the leading cause of mortality in this age group. This scenario placed the country among the ten nations with the highest rates of homicide [9]. It is important to highlight that many other adolescents survive life threatening violent events and are affected by the consequences. Domestic and community violence rates are also high in Brazil [10].

On one hand, it is known that violence exposure increases the risk of development of PTSD symptoms [5]. Furthermore, a large proportion of Brazilian adolescents growing up in urban communities with ongoing violence might also be particularly vulnerable to the development of PTSD due to a combination of other childhood adversities, such as poverty and family violence [11].

On the other hand, not all adolescents will develop PTSD when exposed to adversities and trauma. Epidemiological studies have demonstrated that only a subset of youth who experience violence or other potentially traumatizing events later develop PTSD [12]. For instance, a meta-analysis found that less than 16% of trauma-exposed children and young people experience persistent PTSD [13]. Although there is still much to be learned, researchers have identified risk factors that increase the likelihood of PTSD among those exposed to traumatizing events, such as family functioning, conflicts within relationships with significant others, and low social support. It is important to note that adolescents may perceive conflicts and psychological violence as indicators of lack of family support [14, 15].

Identifying protective factors that may buffer the deleterious effects of risk factors for PTSD development is fundamental. Factors of resilience and social support that coexist with risk factors can moderate or buffer the development of PTSD [16]. Resilient people can adapt to stressful situations, feel less lonely, have better social adaptation skills, and experience greater psychological comfort [17, 18]. Individuals with high-perceived social support are more aware of external resources availability and establish the possibility of being ‘rescued’.

A precise estimate of the proportion of adolescents with PTSD in urban schools in a developing country would allow for a better appraisal of mental health resources. Locally driven epidemiological research is fundamental to support policy, funding, and infrastructure improvements [19]. The present study aimed at investigating: (1) prevalence of PTSD in adolescents from urban schools in low resource communities of São Gonçalo, Brazil; and (2) examine potential associated factors to PTSD, including sociodemographic, individual, family, and community variables.

## Method

### Sample

The study was carried out in the city of São Gonçalo, located in the metropolitan region of the state of Rio de Janeiro, Brazil. The city has a population of over 1 million people, of which one-third are children and adolescents. The city is characterized as a low resource area with low indicators of sewage and urbanization, low per capita income (20% of the population earned $79.4 per month or less in 2016), lack of attendance to school (12.2% of 15–17 years old adolescents out of school), high rates of urban violence (e.g., the homicide rate is 43.9 per 100,000 inhabitants), and child mortality rate was 12.75 per 1000 live births in 2017 [20, 21].

Multi-stage cluster sampling was performed based on a population of 3487 teenagers in 9th grade classes (the last year of fundamental school before entering high school) in public and private schools in 2010. Individuals whose ages were not possible to be accessed or were younger than 13 and above 19 years old were excluded. Sampling followed two stages: (1) schools, with a probable proportional number of 9th grade students, and (2) classes, with the application of the questionnaire to all students present. The sample was designed to obtain proportion estimates with a 95% Confidence Interval (CI) level, an absolute error of 1.6%, and PTSD prevalence in 8% [22]. The estimated sample was 1105 students, and 1129 participated in the study.

A total of 43 public and 30 private schools participated in the study, generally with two 9th grade classes per school. After the school’s approval, the parents signed an informed consent and the adolescents an informed assent. The procedure was explained to the adolescents by experienced researchers and self-administered questionnaires were applied in the classroom. A mental health professional was available to support the students if necessary. The questionnaire was tested in four public schools (51 questionnaires collected) and three private schools (46 adolescents). Students in these schools answered the survey twice within 7 days, to test its reliability.

**Fig. 1:**
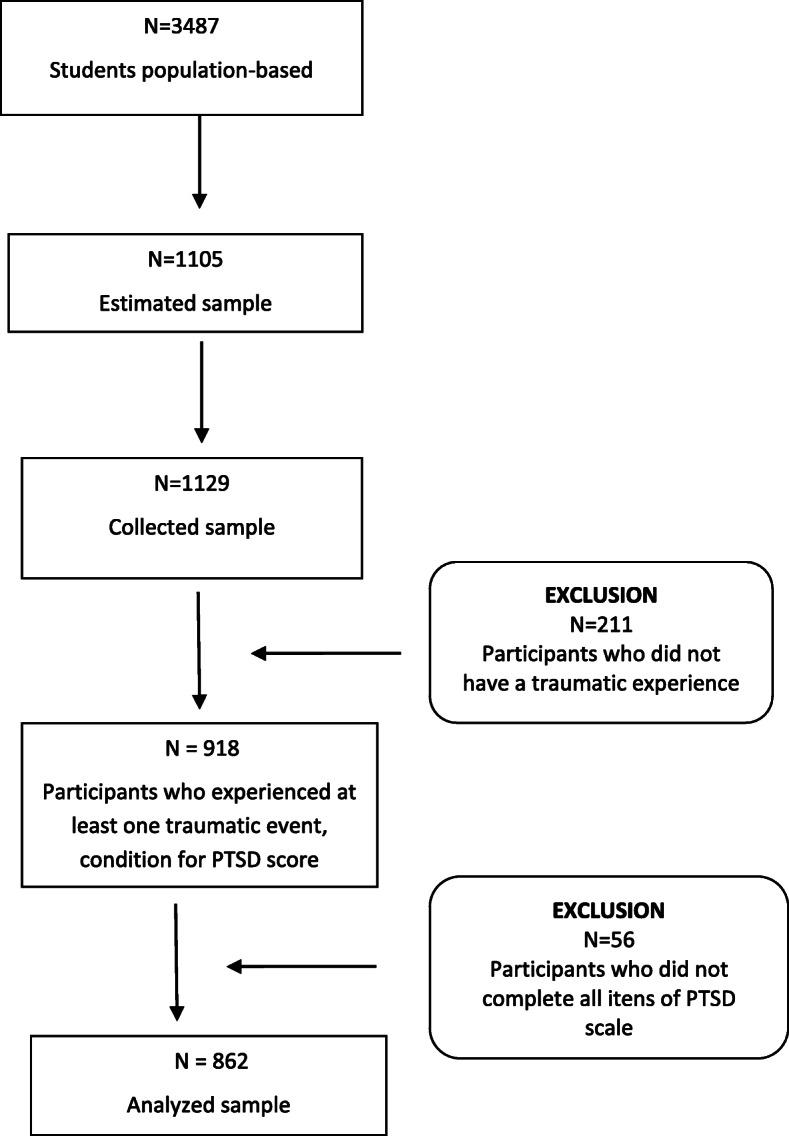
Flowchart of the sample analyzed

Data analysis showed that 918 students experienced at least one traumatic event, essential condition for PTSD according to DSM-IV. In total 862 (65% female and 35% male; *M*age = 15 years old) had complete information about PTSD, and that is the number of adolescents analyzed in this study (Fig. 1). The sample representation was examined by comparing the social stratification in the analyzed sample (*n* = 862) to the initial one obtained (*n* = 1129). No statistical difference was found between the groups, *p* = 0.238).

The research project was authorized by the Ethical Board of the National School of Public Health from Oswaldo Cruz Foundation (CAAE: 0057.0.031.000–09), and written informed consent was obtained from all the adolescents’ and his/her parents/legal guardians.

### Measures

The self-administered structure questionnaire was applied in the classroom with an average duration of 60 min. The questionnaire was composed by validated scales previously used in international studies with Brazilian population:

- *Sociodemographic profile.* Sex, age, skin color, religion, parental education, and income. The income aggregates social stratification groups scored as *upper/middle* and *lower social strata* [23, 24].
- *Family Violence*. *The Conflict Tactics Scale* [25–27] was applied to measure severe physical violence committed by mother and or father against the adolescent, such as kicking, biting, hitting, spanking, burning, strangling, suffocating, threatening with a knife or a gun. At least one positive answer indicates severe physical violence. Satisfactory internal consistency was found for physical violence by the father (Cronbach’s α = 0.69) and by the mother (Cronbach’s α = 0.73) against the child. In the test-retest, most items had a Kappa between 0.62–0.84 (substantial and almost perfect).
- *Psychological violence* was investigated using the *Empirically scaled measures of psychological/verbal control and physical/sexual abuse* of Pitzner e Drummond [28, 29], characterized by 18 items that reflect acts committed by significant people (family included) against the child studied, such as humiliation, criticism, and use of abusive words such as “crazy,” “idiot,” or “stupid”. One point above the standard deviation above the mean was the cut-off point adopted to define adolescents victimized by psychological violence. Excellent internal consistency was showed (Cronbach’s α = 0.93). The Kappa coefficient obtained in almost all items was moderate and substantial (0.42 to 0.68).
- *Sexual violence* was assessed through the question “Did your relationship with your parents involve any kind of sexual experience?”. Answer options were ‘yes’ or ‘no’. This item was developed for this study.
- *School and Community Violence* was measured using the *Self-Reported Offenses* that evaluate the exposure to violence at school and in the community in the past year [30, 31]. The eight items with ‘yes’ or ‘no’ answers include humiliation, threatening, aggression, damage to personal belongings, contact with firearms or knives/cutting instruments, having been robbed, and having had money taken by force in both contexts. The answers were categorized according to absence, presence of one event, or presence of two or more events. The Kuder-Richardson’s coefficient was 0.52 to school violence and 0.57 to community violence, which is acceptable when we consider the low numbers of items in each scale [32].
- *Social support*. The scale *Social Support Battery* developed by Sherbourne and Stewart [33–35] was used. It consists of 19 items and five dimensions: a) emotional (support received through trust, being listened to, sharing concerns/fears, and understanding problems), b) information (receiving suggestions, good advice, information, and desired advice), c) material (someone available to help in case the adolescent has to stay in bed, be taken to the doctor, prepare meals, and help with daily tasks in case of sickness), d) affective (someone demonstrates affection and love, through hugs or other desired forms of caring), and e) positive interaction (someone to have fun together, relax, do nice things and distract the mind). Good internal consistency was showed for affective support (Cronbach’s α = 0.82), positive interaction (Cronbach’s α = 0.85), emotional support (Cronbach’s α = 0.85), and information (Cronbach’s α = 0.84). Due to the small number of items only the material subscale showed lower internal consistency (Cronbach’s α = 0.57). Kappa ranged from 0.099 to 0.744. The scale was used continuously.
- *Social Functional Impairment*. Brief Impairment Scale (BIS) [36, 37] was used to evaluate the overall child and adolescent impairment, based on parents’ vision. It consists of 23 items that include three areas: interpersonal relationship, school functioning, self-care, and self-fulfillment. Scores above 15.5 on the full scale are considered positive for overall functional impairment. Originally, the total scale’s internal consistency varied between 0.81 and 0.88, and between 0.56 and 0.81 in the three subscales [28]. Previous studies reported high convergent and concurrent validity for BIS [34]. Adequate internal consistency was found (Cronbach’s α = 0.72). In the test-retest, most items had a Kappa between 0.42–0.71 (substantial and moderate).
- *Resilience*. The Resilience Scale developed by Wagnild and Young [38–40] was used to evaluate positive psychosocial adaptation levels to adverse life events. It has 25 items, and good psychometric indexes have been obtained for a teenage sample. Adolescents whose items sum was one point below standard deviation from the mean were considered not resilient (73.0 ± 13.0). Adequate internal consistency was found (Cronbach’s α = 0.78). The Kappa coefficient obtained in almost all items was moderate and substantial (0.45 to 0.66).
- *Post-Traumatic Stress Disorder diagnostic*. The UCLA - University of California at Los Angeles Post-Traumatic Stress Disorder Reaction Index for DSM-IV for adolescents was used [41, 42]. The instrument is divided into three parts: (1) 14 items to assess traumatic life events. The item regarding earthquake was replaced by landslide to adapt to the Brazilian context; (2) 13 items to assess subjective characteristics of exposure to trauma; and (3) 22 items that provide an assessment of PTSD symptoms during the last month. Response options on the Likert scale are: never, rarely, sometimes, often, most of the time, with scores ranging from 0 to 4. The items evaluate the presence of intrusive memories, persistent avoidance, and persistent symptoms of increased excitability (distributed among B, C, and D criteria). They are evaluated by the severity criterion resulting from the sum of 17 items that meet the DSM-IV criteria (reaching a score of 38 or higher configures PTSD), proven by the sensitivity and specificity achieved according to Steinberg et al. [41]. This instrument was submitted to the cross-cultural adaptation process for the Brazilian adolescent population [43], with α = 0.866 for the items that compose the severity score (criteria B, C, and D) and significant correlations with the depression scale [44] (Spearman’s rho = 0.405, *p* < 0.001). Good internal consistency was showed (Cronbach’s α = 0.89). Kappa referred to the majority of items of traumatic life events ranged from 0.62 to 0.88 (substantial and almost perfect) and for PTSD symptoms between 0.40 to 0.71 (substantial and moderate).

### Statistical analyses

First of all, traumatic life events were evaluated according to sex. Second, data was organized in sociodemographic, individual, family, and community blocks and investigated regarding its association with PTSD diagnostic (chi-square and Fisher’s exact test), with a significance level of 5%. The frequencies were corrected for the sample weights, the odds ratios were estimated through logistic regression, and the point estimates were also corrected. The sampling plan was inserted in the analysis for accuracy correction.

## Results

A total of 7.8% of adolescents surveyed presented PTSD in a clinical level using the UCLA score. The most frequent events reported were related with experiences within the community: seeing a dead body (61%); hearing about the violent death or serious injury of a loved one (51%); being in a place where armed conflicts/shootings were happening (43%), and seeing someone being beaten up, shot at or killed (43%). Regarding violence at home, reports included seeing a family member being hit, punched or kicked very hard (28%), and being hit, punched, or kicked very hard at home (24%). As can be seen in Table 1, when comparing the differences of frequencies of experiencing traumatic events to girls, boys significantly reported more often seeing a dead body (*p* < .001), experiencing armed conflicts (*p* < .05), having painful and scary medical treatment in a hospital due to sickness or bad injury (*p* < .001), being in a serious accident (*p* < .001), and serious community violence by being beaten up, shot at or threatened where they live (*p* < .001). Girls had significantly more experiences with hearing about the violent death or severe injury of a loved one when compared with boys (p < .001), and family violence by seeing a family member being hit, punched or kicked very hard (*p* < .05) (Table 1).

**Table 1:** Prevalence of traumatic life events according to the sex of adolescents from public and private schools in Rio de Janeiro/Brazil (*n*=862)

Variables	female (%)	male (%)	***p***.value*
Being in a landslide^a^ that badly damaged the building you were in (*n* = 858)	5.9 (*n* = 36)	9.8 (*n* = 28)	.067
Being in another kind of disaster, like a fire, tornado, flood or hurricane (*n* = 856)	14.8 (*n* = 90)	17.4 (*n* = 51)	.404
Being in a bad accident, like a very serious car accident (*n* = 859)	11.3 (*n* = 63)	19.8 (*n* = 60)	**.000**
Being in place where armed conflict (shootings) was going on around you (*n* = 859)	40.9 (*n* = 230)	49.2 (*n* = 144)	**.017**
Being hit, punched, or kicked very hard at home (DO NOT INCLUDE ordinary fights between brothers and sisters) (*n* = 858)	24.2 (*n* = 137)	24.2 (*n* = 70)	.989
Seeing a family member being hit, punched or kicked very hard at home (DO NOT INCLUDE ordinary fights between brothers and sisters) (*n* = 855)	29.6 (*n* = 164)	27.3 (*n* = 79)	.049
Being beaten up, shot at or threatened to be hurt badly in your town (*n* = 859)	10.9 (*n* = 65)	22.6 (*n* = 62)	**.001**
Seeing someone in your town being beaten up, shot at or killed (*n* = 853)	41.9 (*n* = 229)	44.5 (*n* = 135)	.442
Seeing a dead body in your town (*n* = 858)	56.5 (*n* = 314)	70.7 (*n* = 209)	**.000**
Having an adult or someone much older touch your private sexual body parts when you did not want them to (*n* = 860)	10.2 (*n* = 60)	9.6 (*n* = 28)	.760
Hearing about the violent death or serious injury of a loved one (*n* = 856)	54.6 (*n* = 299)	43.6 (*n* = 140)	**.001**
Having painful and scary medical treatment in a hospital when you were very sick or badly injured (*n* = 859)	13.8 (*n* = 81)	21.9 (*n* = 65)	**.003**
Other traumatic situations (*n* = 851)	22. (*n* = 117)	23.7 (*n* = 64)	.692

*p.values from Pearson’s chi-squared test statistics;

^a^Earthquake was replaced to landslide to adapt to the Brazilian context

Table 2 shows the prevalence of PTSD according to sociodemographic variables. Age was significantly associated with PTSD outcome (OR, 1.447, [95% CI, 1.04–2.00]): the increase of one year corresponds to an increase of almost 45% in the chance of having PTSD. Sex, skin color, social stratum, religion, and parents’ schooling had no significant association with the studied disorder.

**Table 2:** Prevalence of PTSD according to sociodemographic variables (*n*=862)

Variables		% PTSD	OR	IC95%
**Sociodemographic variables**				
Sex	Female (*n* = 561)	9.2	1.912	0.787–4.643
	Male (*n* = 301)	5.0	–	–
Skin color	Indigenous (*n* = 38)	2.1	0.260	0.017–4.073
	Black (*n* = 491)	8.1	1.074	0.503–2.292
	White (*n* = 322)	7.6	–	–
Age^1^	13–19 (*n* = 870)	–	1.447	**1.043–2.007**
Religion	No (*n* = 200)	7.5	0.942	0.378–2.35
	Yes (*n* = 657)	7.9	–	–
Social strata	Lower social strata (*n* = 192)	6.8	1.081	0.383–3.049
	Upper/middle (*n* = 297)	6.3	–	–
Mothers’ formal education	Complete Elementary School/Incomplete High School (*n* = 123)	8.9	0.530	0.107–2.638
	Complete High School/Incomplete College (*n* = 267)	6.8	0.768	0.19–3.096
	Complete College (99)	2.9	0.708	0.152–3.307
	Do not read/Incomplete Elementary School (*n* = 152)	7.5	–	–
Father’s formal education	Complete Elementary School/ Incomplete High School (*n* = 108)	4.8	0.530	0.107–2.638
	Complete High School/Incomplete College (*n* = 212)	6.8	0.768	0.190–3.096
	Complete College (*n* = 85)	6.3	0.708	0.152–3.307
	Do not read/Incomplete Elementary School (*n* = 125)	8.6	–	–

^1^Age was evaluated by the increase of one year

Individual attributes such as social functional impairment (OR, 4.82, [95% CI, 1.78–13.10]) and resilience (OR, 0.46, [95% CI, 0.26–2.11]) were also investigated, and only the first one was associated with PTSD diagnostic. The results show that adolescents with signs of impairment in social functioning were 4.8 times more likely to have PTSD than those without malfunction. Regarding the family environment, severe physical violence perpetrated by the mother (OR, 2.80, [95% CI, 0.79–9.93]) and psychological violence practiced by significant people (OR, 3.96, [95% CI, 1.89–8.31]) might increase the chance of having a PTSD diagnostic. The ability to request and receive support was not significantly associated with the presence of PTSD in general. Experiencing one (OR, 2.86, [95% CI, 1.18–6.90]), two or more (OR, 3.52, [95% CI, 1.47–8.40]) event types of community violence were significantly associated with PTSD in the group studied (Table 3).

**Table 3:** Prevalence of PTSD according to individual, family and community variables (*n*=862)

Variables		% PTSD	OR	IC95%
**Individual variables**				
Social functional impairment	With impairment (*n* = 504)	11.3	4.825	**1.776–13.105**
	Without impairment (*n* = 358)	2.6	–	–
Resilience	Resilient (*N* = 554)	8.9	–	–
	Not resilient (*n* = 82)	6.7	0.742	0.261–2.107
**Family variables**				
Severe physical violence of the mother (last year)	Presence (*N* = 153)	13.5	2.794	**0.786–9.929**
	Absence (*N* = 600)	6.6	–	–
Severe physical violence of the father (last year)	Presence (*N* = 66)	16.2	0.625	0.039–10.028
	Absence (*N* = 656)	6.5	–	–
The relationship with the parents involved a sexual experience	Yes (*N* = 16)	5.1	0.625	0.039–10.028
	No (*N* = 842)	7.8	–	–
Psychological violence	Presence (*N* = 123)	19.2	3.96	**1.887–8.309**
	Absence (*N* = 632)	5.7	–	–
Affective support (Social support)	Score	–	0.985	0.968–1.003
Positive interaction (Social support)	Score	–	0.988	0.969–1.006
Emotional support (Social support)	Score	–	0.985	0.969–1.003
Information support (Social support)	Score	–	0.981	0.964–0.999
Material support (Social support)	Score	–	0.984	0.963–1.005
**Community Variables**				
School Violence	2 or more (*N* = 147)	11.3	2.362	0.889–6.27
	One (*N* = 198)	9.7	2.007	0.844–4.77
	None (*N* = 476)	5.1	–	–
Community Violence	2 or more (*N* = 169)	13.4	3.518	**1.473–8.402**
	One (*N* = 176)	11.2	2.856	**1.181–6.905**
	None (*N* = 485)	4.2	–	–

## Discussion

This study addressed an important gap in trauma research in LMIC by examining factors associated with PTSD in the sample of Brazilian adolescents. This study investigated the prevalence of PTSD in adolescents from urban schools in one low resource city in Brazil. Key findings were as following: (1) PTSD prevalence was relatively high, 7.8% of the adolescents fulfilled the diagnostic criteria; (2) community violence was often reported, e.g., more than half of males and females have seen a dead body in their neighborhood, and almost half have witnessed shootings, someone being beaten up, shot or killed; (3) boys experienced significantly more severe traumatic events than girls, including community violence; (4) girls experienced significantly more events of family violence than boys; and (5) PTSD diagnostic was significantly associated with age, social functional impairment, severe physical violence by mothers, psychological violence by significant people, and experiencing a higher number of community violence events.

None of the sociodemographic variables were significantly associated with PTSD except for age, which can be explained by more exposure to adversities by older adolescents. The number of exposures to adversities has been reported as an essential risk factor for PTSD development, an effect called building block [45, 46]. Adolescents might be at peak age for trauma exposure from16–20 years of age [47], possibly due to less supervision and parents’ social support. Current models on PTSD etiology describe symptoms severities mainly as a linear function of cumulative adverse childhood experiences [48]. It may also explain the association of PTSD with the experience of two or more events of community violence.

It is interesting to note that studies report a higher incidence of PTSD in girls [49, 50], which is, in part, explained by their greater exposure to sexual violence, the differences in coping style, and the more significant presence of anxiety problems. In contrast, boys are more associated with the idea of aggressiveness, which would lead them to suppress symptoms or to have cognitive strategies related to the trauma [49–51]. In the present study there was no significant difference between males and females, regarding sexual abuse and PTSD. The high prevalence of community-based violence events may overlap significantly among boys, which may balance the psychopathological effects of events among boys and girls.

In line with previous research [52], the data shows the association between PTSD and social functional impairment. Adolescents with PTSD who have experienced prolonged and repeated stressors may also present self-destructive behavior, substance abuse, intermittent rage, or aggressive outbursts. They may also present feeding problems, feelings of guilt, and substance abuse. Changes in conceptions about their identity, future, and security may affect the short-lived feeling that may be characterized by the belief that they will not establish a career or form a family, among other ordinary young people’s expectations [53]. Alisic et al. [13] explain that traumatic events such as war, violence, and terrorism have a higher impact on the onset of PTSD. They are generally chronic, erode social support, lead to more self-blame or other maladaptive cognitions, and represent a ‘betrayal’ of trust that affects social functioning.

Social support and resilience also showed no association with PTSD. This can be hypothetically explained by the high prevalence of social support in the interviewed adolescents (data not shown). A potential elucidation for the lack of relationship between resilience and mental health might be more complicated than the simple absence of PTSD symptoms [54]. Another possible explanation is that adolescents with high resilience levels might buffer the harmful effects of childhood abuse and neglect, but not community violence [17]. For instance, a study with veterans with PTSD found that higher resilience was associated with more intact social functioning regardless of the severity of PTSD [55]. Probably, a complex interaction between (epi)genetic and environmental factors also contribute to the relationship between resilience and PTSD. Exposure to high domestic and community violence levels is associated with epigenetic changes in adolescents living in low-resource areas, and its effects might be observed in following generations [45]. Further studies are needed to better understand the connections between resilience and PTSD.

The experience of abuse is a significant and independent predictor of poor mental health outcomes such as anxiety, depression, and PTSD [56, 57]. Individuals who experience psychological aggression are significantly more likely to have relationship dissatisfactions and suffer physical victimization [58]. Notably, the same characteristics that increase the likelihood of exposure to violence may also serve to increase vulnerability for developing PTSD symptoms in response to violence exposure. Interestedly, maternal physical violence was associated with PTSD, in contrast to father physical violence. This finding suggests that the relation of maternal versus paternal physical violence work differently for PTSD in adolescents. Also, Moretti and Craig [59] found no direct association between paternal abuse and depressive symptoms, whereas maternal abuse was associated with adolescents’ reports of depressive symptoms. These authors explained that the effects of maternal maltreatment may be a function of the roles that mothers play as primary caregivers and attachment figures and are primary sources of emotional support [60, 61].

One of the strengths of the study is to assess, comprehensively, a sizeable representative school sample in a low-resource area with high rates of different types of violence and lack of access to public mental health. More locally driven strategies for preventing risk factors for PTSD are needed. Services designed specifically for caring for young people’s mental health in developing countries were reported very scarce in comparison to developed countries [19]. We expect to contribute to evidence-based public policies to promote adolescents’ well-being. Some limitations need to be pointed out: (1) due to the cross-sectional design, we were unable to determine directionality or causality within the variables; (2) the self-reported survey data may be subject to recall and reporting biases, even though a high consistency between the measures and the results exists, (3) the non-inclusion in the analysis of concomitant multiple exposures to traumatic experiences, which can help in understanding the relationships between trauma, resilience and coping abilities.

## Conclusion

High community violence, family violence, differences in individual life trajectories for boys and girls, age, and social functional impairment are factors associated with PTSD that need to be better understood and prevented. Designing preventive strategies and offering appropriate treatment for children and adolescents with PTSD is challenging for countries, especially those with a lower degree of social and economic development. Researches in this area typically emphasize multiple levels or domains of influence and symptoms of PTSD rarely disappear spontaneously in children and adolescents. Brazilian children and adolescents as well as others living in less developed countries have an insufficient supply of mental health services, even in large urban centers. It is urgent to move efforts to prevent family and community violence and offer this population treatment with a mental health team in the first stages of life to reduce and relieve suffering allowing thus a better future.

## Supplementary Information

**Additional file 1.** About the question used for evaluation of information for sexual violence: Does your relationship with your parents involve any kind of sexual experience? () yes () no.

## Data Availability

The datasets generated and or analyzed during the current study are not publicly available due to ethical restrictions and personal data protection, but are available from the corresponding author on reasonable request.

## Abbreviations

CI: Confidence Interval
DSM-IV: Diagnostic and Statistical Manual of Mental Disorders
OR: Odds ratio
PTSD: Posttraumatic stress disorder
US: United States
