# Supplementary material for: Posttraumatic stress disorder among adolescents in Brazil: a cross-sectional study
Source: BMC Psychiatry. 2021 Feb 5;21:75. doi: 10.1186/s12888-021-03062-z (PMC7866458; doi:10.1186/s12888-021-03062-z)
Supplement: Supplementary file 1 — Additional file 1. About the question used for evaluation of information for sexual violence: Does your relationship with your parents involve any kind of sexual experience? () yes () no. [file 12888_2021_3062_MOESM1_ESM.docx]

**Question used for evaluation of information for sexual violence**

Does your relationship with your parents involve any kind of sexual experience?

( ) yes ( ) no
